# Polychlorinated Biphenyls and Cancer: Are Telomeres to Blame?

**DOI:** 10.1016/j.ebiom.2015.12.011

**Published:** 2015-12-15

**Authors:** Shahinaz M. Gadalla, Gabriella Andreotti

**Affiliations:** a Clinical Genetics Branch, Division of Cancer Epidemiology and Genetics, National Cancer Institute, Bethesda, Maryland, United States; b Occupational and Environmental Epidemiology Branch, Division of Cancer Epidemiology and Genetics, National Cancer Institute, Bethesda, Maryland, United States

The accumulating epidemiological evidence of elevated cancer risk and mortality in individuals exposed to polychlorinated biphenyls (PCBs) led to their recent classification as human carcinogen by the International Agency for Research on Cancer (IARC) (International Agency for Research on Cancer (IARC), 2015). However, the mechanisms by which PCBs are linked to cancer are still unclear.

In this issue, Scinicariello and Buser (Scinicariello, 2015) conducted a study of the association between leukocyte telomere length (LTL) and PCB blood levels in a nationally representative sample of the civilian US adult population using data from the National Health and Nutrition Examination Survey (NHANES). The authors showed that higher PCB blood levels were associated with longer LTL, and hypothesized that there could be a link to PCB-related carcinogenesis. A separate study reported similar relationships between LTL and PCB blood levels in a subset of NHANES participants (Mitro et al., 2015). However, a small study of healthy Koreans (Shin et al., 2010) showed that this relationship was only present at low PCB levels. In contrast, short LTL has been associated with a number of environmental or occupational exposures including particulate matter, black carbon, benzene, toluene, polycyclic aromatic hydrocarbons, N-nitrosamines, pesticides, and lead (reviewed in Zhang et al., 2013), all of which may contribute to PCB blood levels. Telomere shortening in response to chemical exposures may be explained, at least in part, by the induction of an oxidative stress DNA damage response (Zhang et al., 2013).

Telomeres, the long (TTAGGG)^n^ nucleotide repeats and an associated protein complex at chromosome ends, are essential for maintaining chromosomal stability. They shorten with each cell division and therefore are markers for cellular replicative capacity, cellular senescence, and aging. Telomere shortening and chromosomal instability has been described at early stages of carcinogenesis, suggesting a role of telomere dysfunction in cancer initiation (Ferguson et al., 2015).

Cancer epidemiology studies have shown relationships with short LTL, but differences by cancer site and study design were noted. In a meta-analysis of 27 reports (Wentzensen et al., 2011), short LTL was associated with cancer risk, primarily in case–control studies (OR = 2.9 in case–control studies vs. 1.2 in prospective studies). This suggests that short LTL is a disease marker rather than a risk factor.

Recently, a large study linked long LTL to melanoma risk (Iles et al., 2014). The spectrum of cancers associated with long LTL is not clear yet, but suggested to include lung and Non-Hodgkin's lymphoma (NHL). It is of interest that cutaneous melanoma and possibly NHL are the main cancers associated with excess risk in PCBs exposed individuals (International Agency for Research on Cancer (IARC), 2015), suggesting a possible contribution of LTL in PCB-related carcinogenesis.

The large sample size, the national representation, and the wealth of collected information NHANES offers are among the strengths of this study. On the other hand, the cross-sectional design restricted its ability to establish a causal inference between LTL, PCB exposure, and cancer risk. The one-time measurement of PCB blood level and LTL may not reflect the full spectrum of the association. Information on the duration and dose of exposure to PCBs would have been useful (reviewed in Zhang et al., 2013) Short-term exposure to ambient particulate matter was associated with long LTL, while longer exposures were associated with short LTL. Exposure dose in relation to LTL was noted in other environmental exposures. For example, exposure to low dose of arsenic resulted in telomerase overexpression and telomere elongation in cord blood cells, while exposure at large doses suppressed telomerase, shortened telomeres, and induced apoptosis. A recent study (Andreotti et al., 2015) evaluating the association between LTL and pesticide exposure suggested a differential effect on LTL in long-term versus recent exposures for certain pesticides.

While LTL may present a good surrogate for telomere length in other tissues, it is not clear how good of a surrogate it is for tissues affected by chemical exposures. Measuring telomere length in organs directly affected by PCB exposure, and comparing this to LTL may explain the role telomeres may play in PCB-related carcinogenesis. Also, LTL measurement obtained by quantitative polymerase chain reaction (qPCR) assay may be affected by the cell composition of the sample since telomere length differs by peripheral blood cell subtype. Using a leukocyte cell-type specific TL measurement assay such as flow FISH may provide better insights into such association. Important considerations in epidemiological studies of telomere length are discussed in details elsewhere (Bodelon et al., 2014).

In conclusion, the mechanism behind PCB-related carcinogenesis is still unknown. The similarity between cancer sites associated with long LTL, and cancers linked with PCBs provides an interesting hypothesis to test. A large prospective study with serial LTL and PCBs level measurements is required to better understand this relationship.
